# Supplementary figures and images for: In vivo single-cell high-dimensional mass cytometry analysis to track the interactions between Klebsiella pneumoniae and myeloid cells
Source: PLoS Pathog. 2024 Apr 5;20(4):e1011900. doi: 10.1371/journal.ppat.1011900 (PMC11023633; doi:10.1371/journal.ppat.1011900)

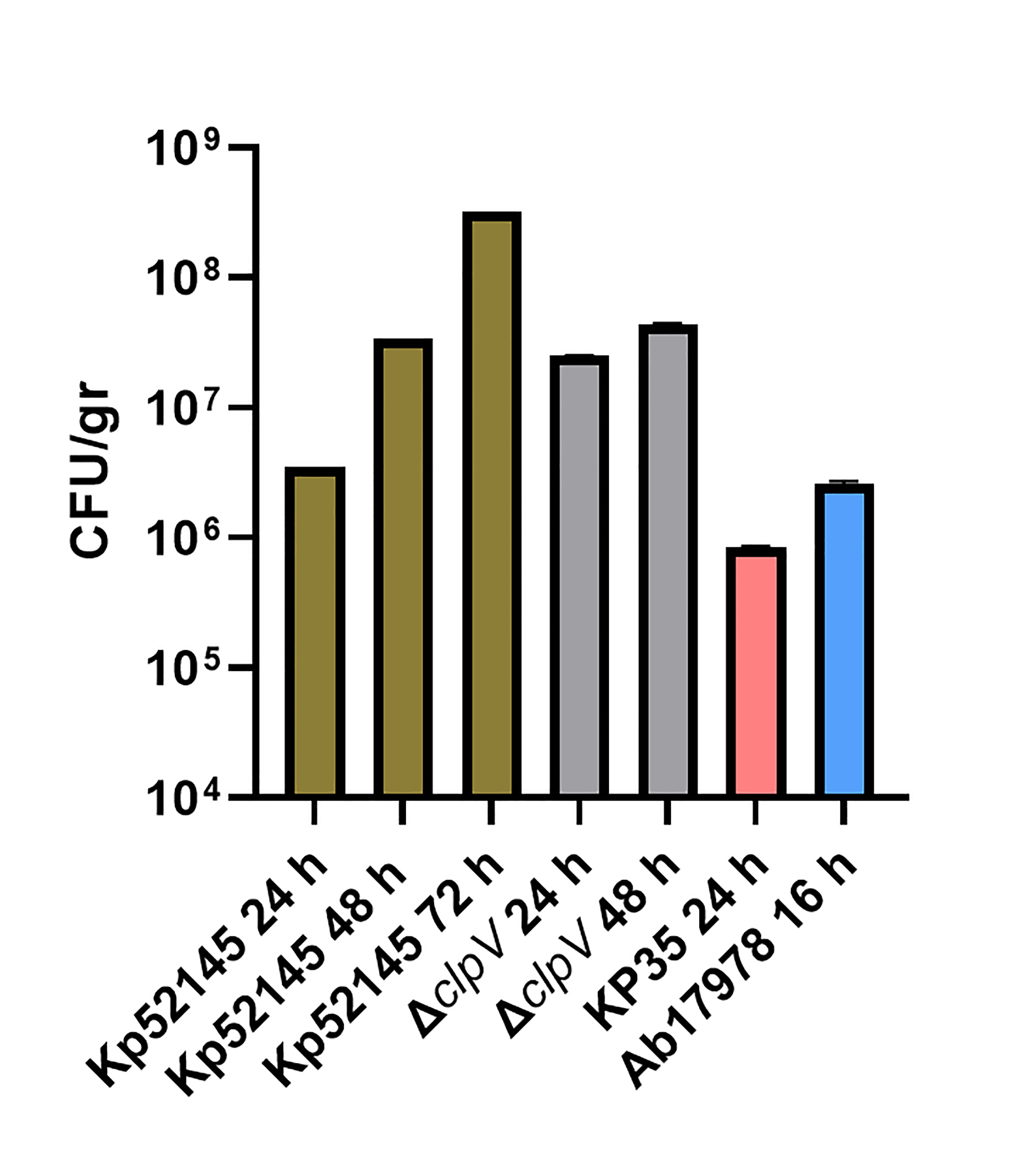

Supplement: S1 Fig — CFU per gr of lung of mice infected with Kp52145 for 24, 48 and 72 h, the T6SS clpV mutant (ΔclpV) for 24 andf 48 h, KP35 for 24 h, and A. baumannii ATCC17978 (Ab17978) for 16 h. Results (mean and SD) are based on data from three mice per group. (TIF) [file ppat.1011900.s001.tif]

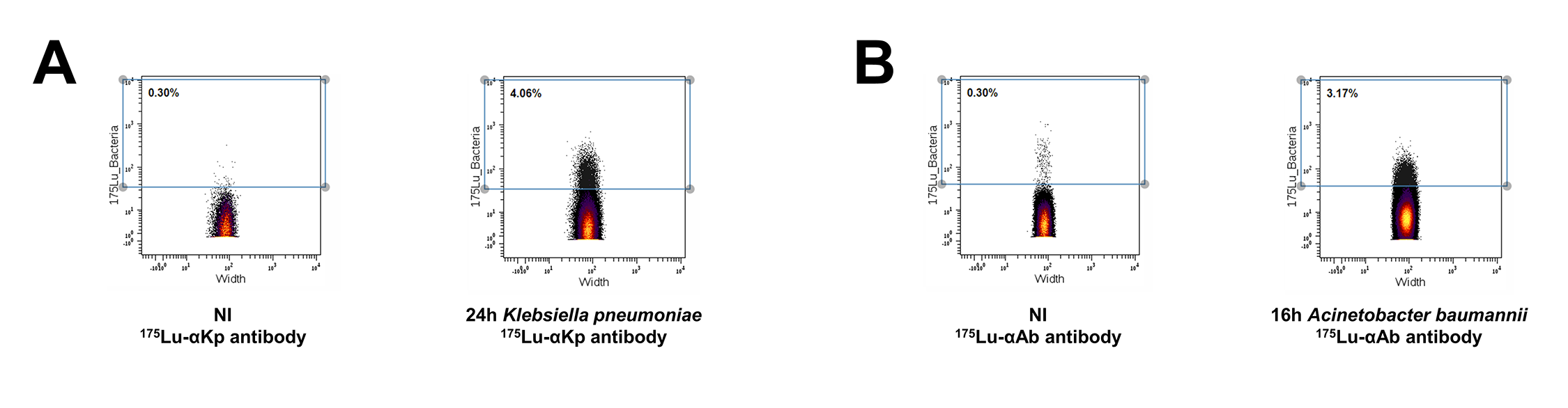

Supplement: S2 Fig — A. Manual gating approach to identify cells positive upon incubation with the anti-K. pneumoniae antibody. B. Manual gating approach to identify cells positive upon incubation with the anti-A. baumannii antibody. (TIF) [file ppat.1011900.s002.tif]

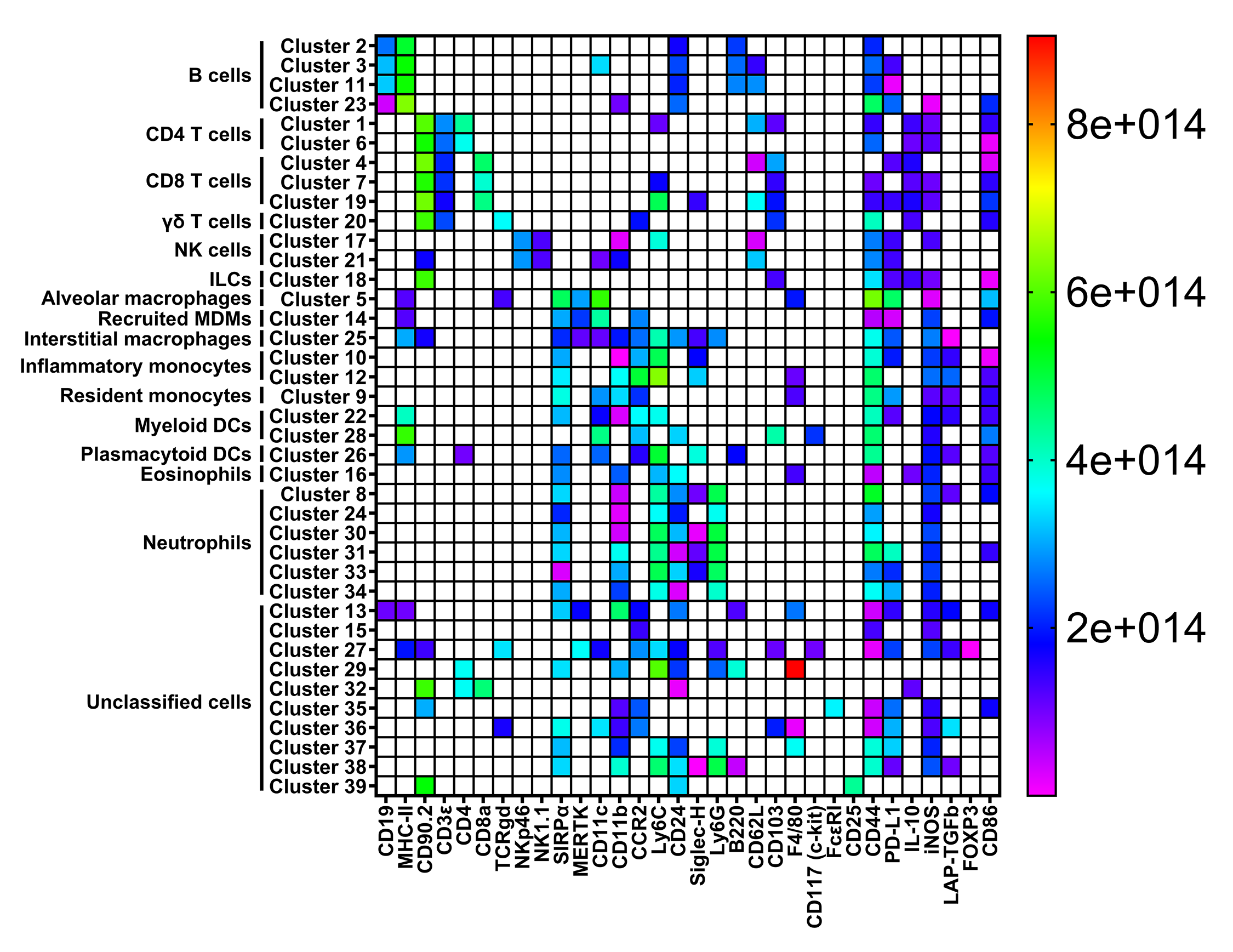

Supplement: S3 Fig — Heatmap showing relative signal intensities of the indicated markers on the populations and subpopulations found in this study. The heatmap is coloured based on signal intensity of the indicated markers. Results are based on data from three mice per group. (TIF) [file ppat.1011900.s003.tif]

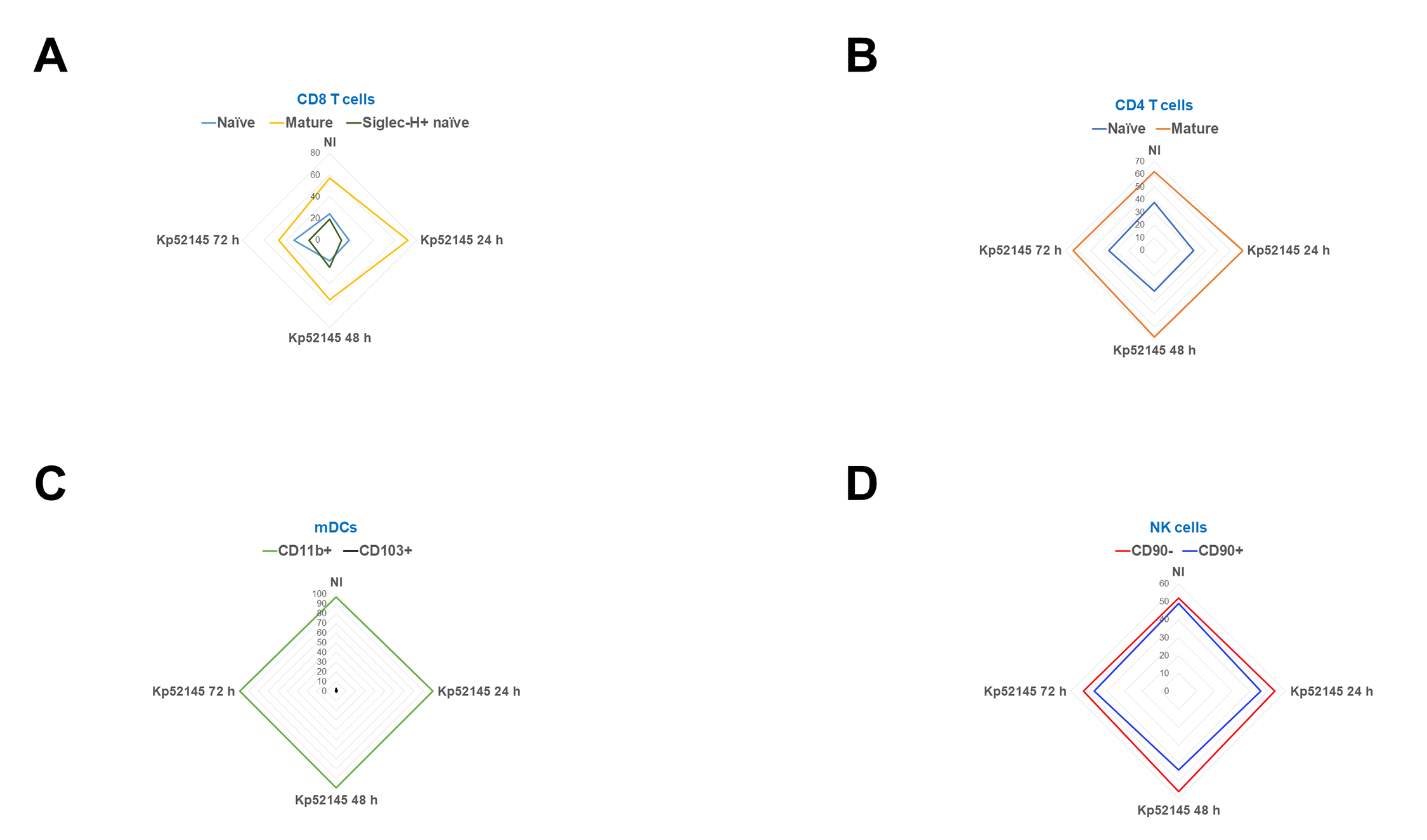

Supplement: S4 Fig — Radar plots show the percentage of subpopulations of A. CD8 T cells, B. CD4 T cells, C. mDCs, and D. NK cells in the lungs of wild-type mice non-infected (ni) or infected with Kp52145 for 24, 48 and 72 h. (TIF) [file ppat.1011900.s004.tif]

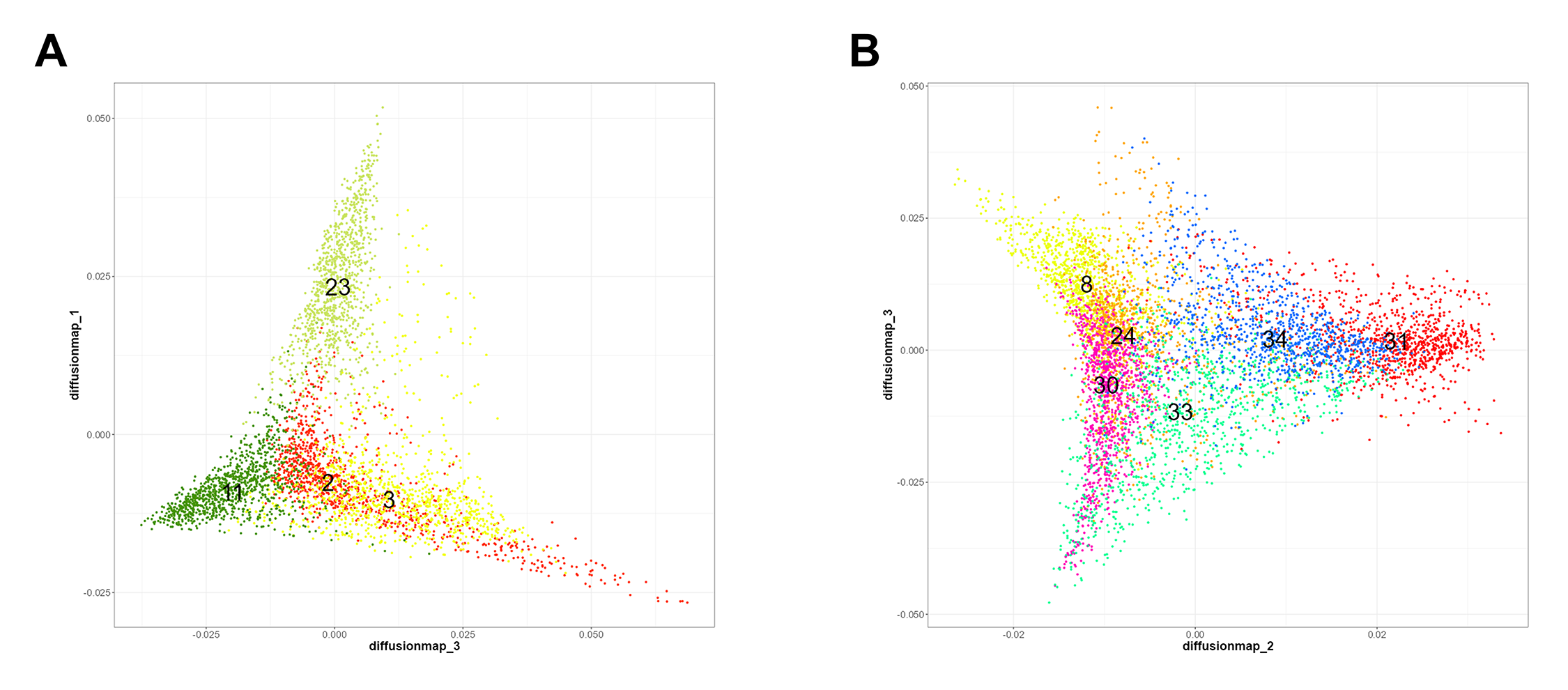

Supplement: S5 Fig — A. Diffusion map of the data set depicting B cell maturation. B. Diffusion map of the data set depicting neutrophil differentiation. (TIF) [file ppat.1011900.s005.tif]

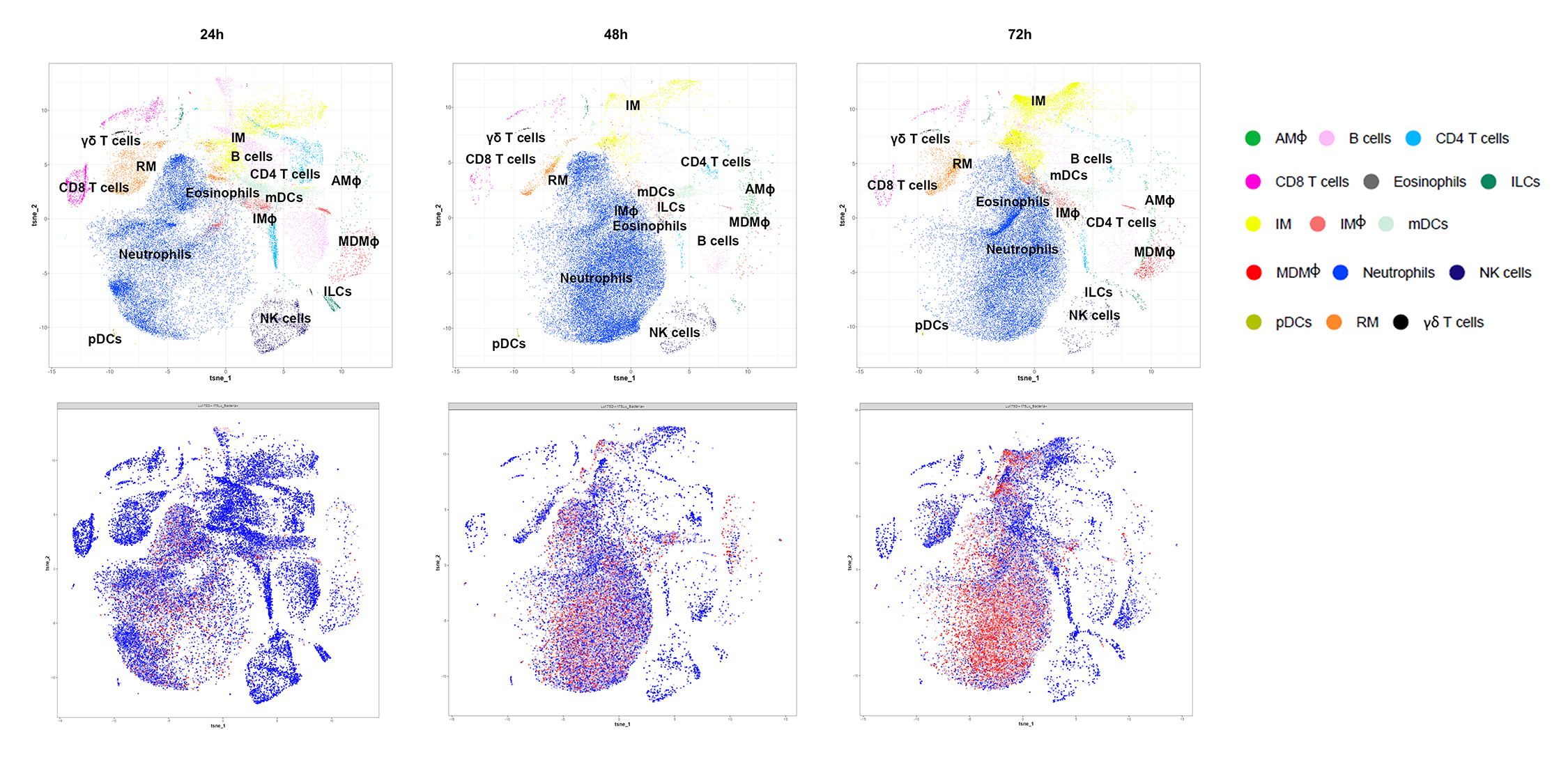

Supplement: S6 Fig — t-SNE analysis of the populations of lung immune cells in the lungs of wild-type mice infected with Kp52145 for 24, 48 and 72 h. In red it is marked the t-SNE analysis of the Klebsiella marker, indicating presence of K. pneumoniae within the identified immune cells. Results are based on data from three mice per group. (TIF) [file ppat.1011900.s006.tif]

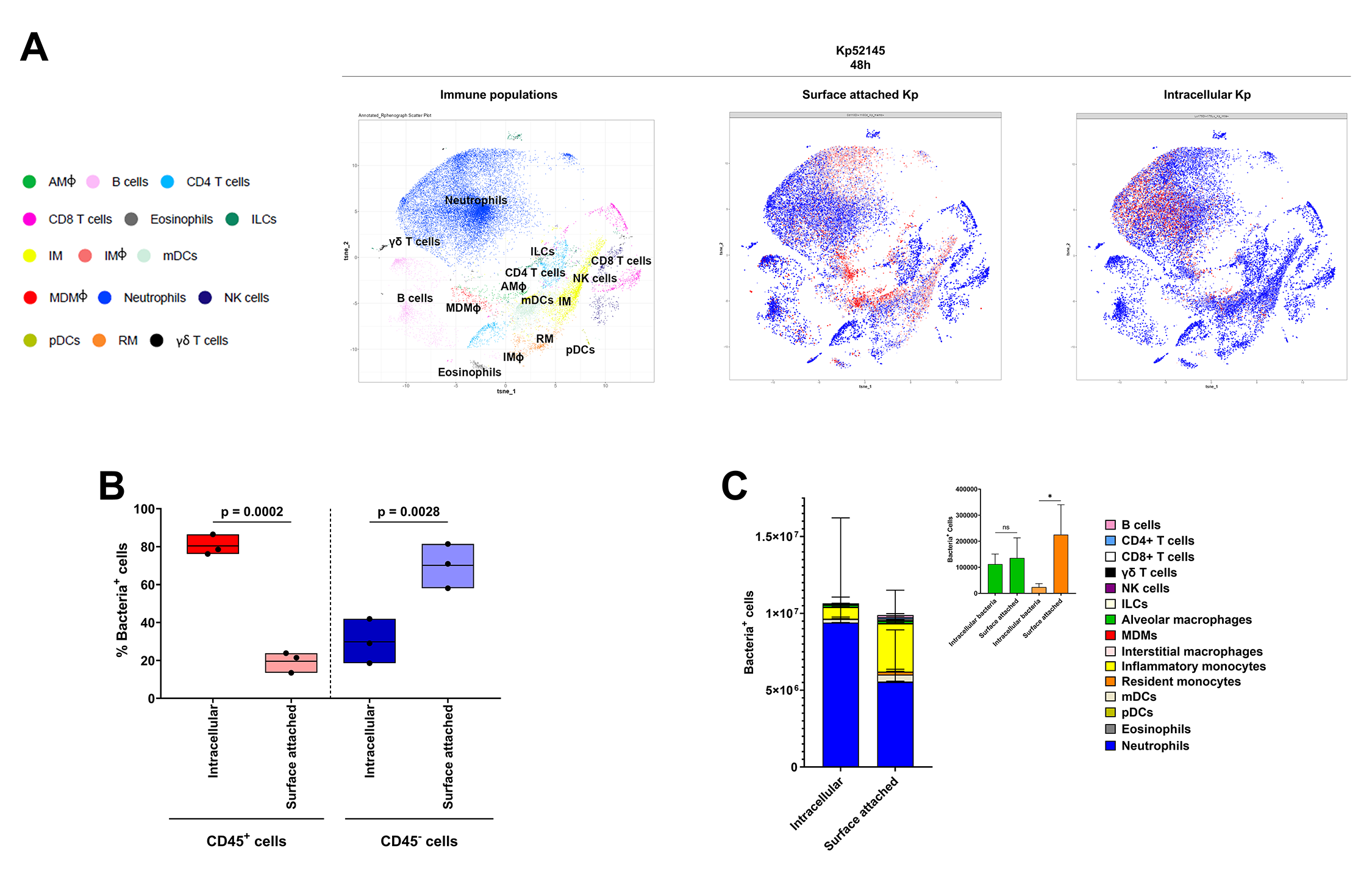

Supplement: S7 Fig — A. t-SNE analysis of the populations of lung immune cells in the lungs of wild-type mice infected with Kp52145 for 48. In red it is marked the t-SNE analysis of the Klebsiella marker, indicating presence of K. pneumoniae surface attached or intracellular within the identified immune cells. Results are based on data from three mice per group. B. Percentage of CD45+ and CD45- cells with surface attached or intracellular bacteria in the lungs of wild-type mice infected with Kp52145 for 48. C. Number of cells with surface attached or intracellular bacteria in the lungs of wild-type mice infected with Kp52145 for 48. Inset depicts the number of alveolar macrophages and resident monocytes with surface attached or intracellular bacteria. Results are based on data from three mice per group. (TIF) [file ppat.1011900.s007.tif]

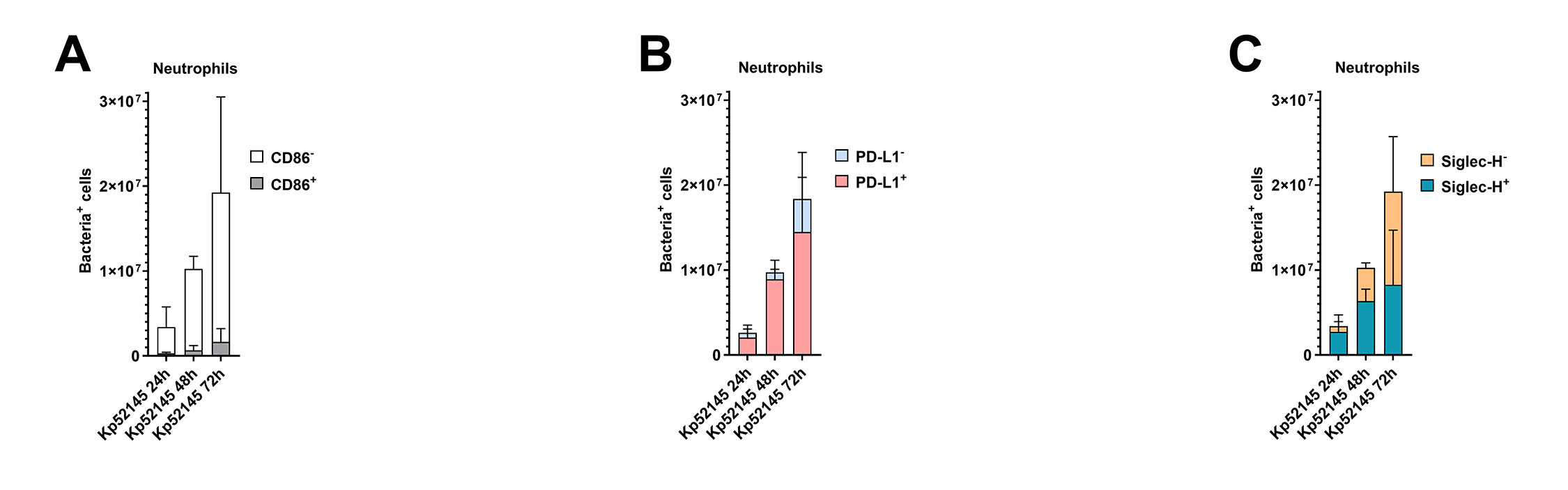

Supplement: S8 Fig — A. Number of infected CD86+ and CD86- subpopulations of neutrophils in the lungs of wild-type mice infected for 24, 48 and 72 h with Kp52145. Results are based on data from three mice per group. B. Number of infected PD-L1+ and PDL-L1- subpopulations of neutrophils in the lungs of wild-type mice infected for 24, 48 and 72 h with Kp52145. Results are based on data from three mice per group. C. Number of infected Siglec-H+ and Siglec-H- subpopulations of neutrophils in the lungs of wild-type mice infected for 24, 48 and 72 h with Kp52145. Results are based on data from three mice per group. (TIF) [file ppat.1011900.s008.tif]

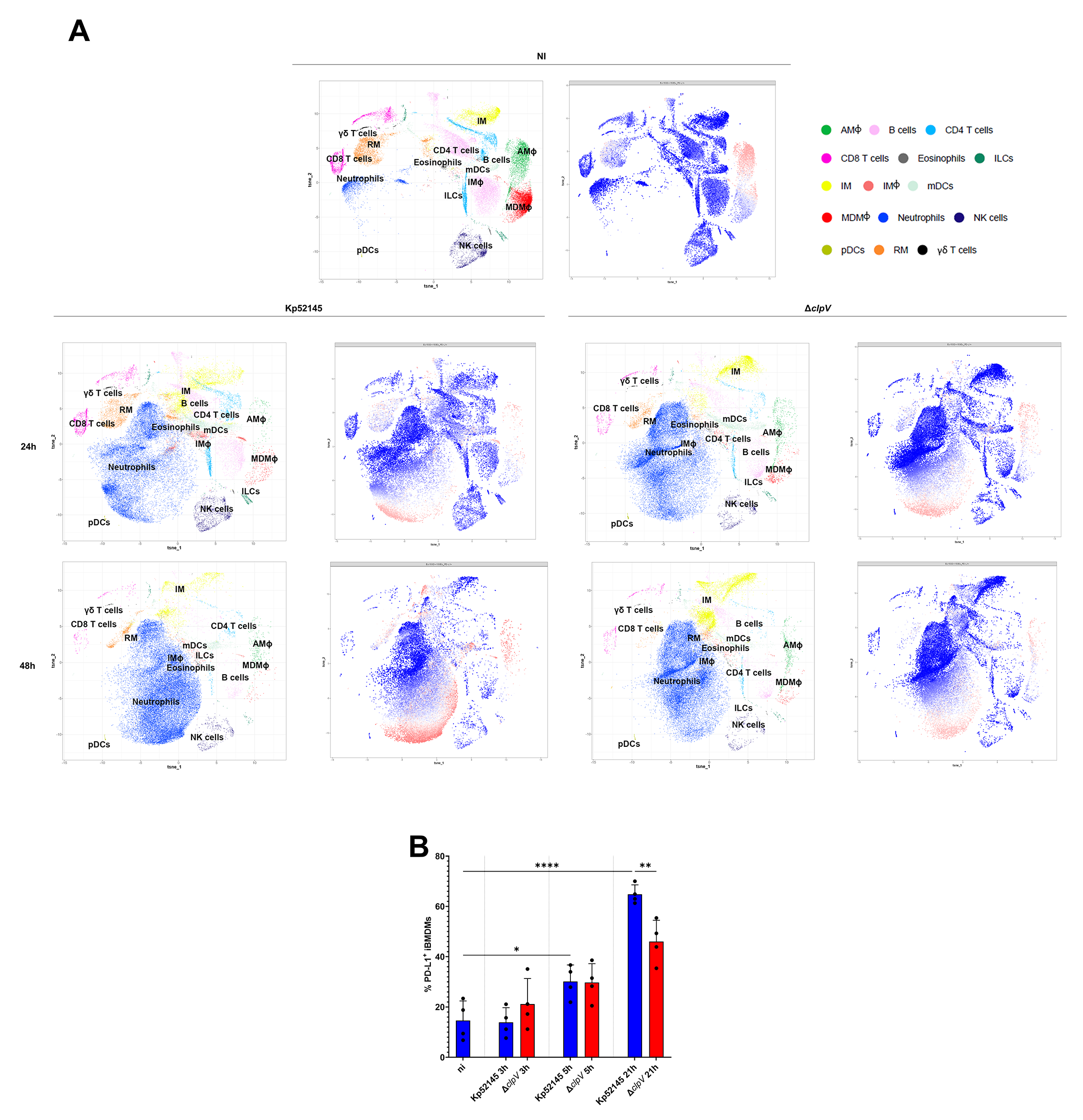

Supplement: S9 Fig — A. t-SNE analysis of the populations of lung immune cells in the lungs of wild-type mice non-infected (NI), infected with Kp52145, and the T6SS clpV mutant (ΔclpV) for 24 and 48 h. In red it is marked the t-SNE analysis of the PD-L1 marker, indicating presence of the marker within the identified immune cells. Results are based on data from three mice per group. B. PD-L1 levels in iBMDMs from wild-type mice infected with Kp52145 and the T6SS clpV mutant (ΔclpV) for the indicated time. ****p ≤ 0.0001, **p ≤ 0.01, *p ≤ 0.05; for the indicated comparisons using one-way ANOVA with Bonferroni contrast for multiple comparisons test. (TIF) [file ppat.1011900.s009.tif]

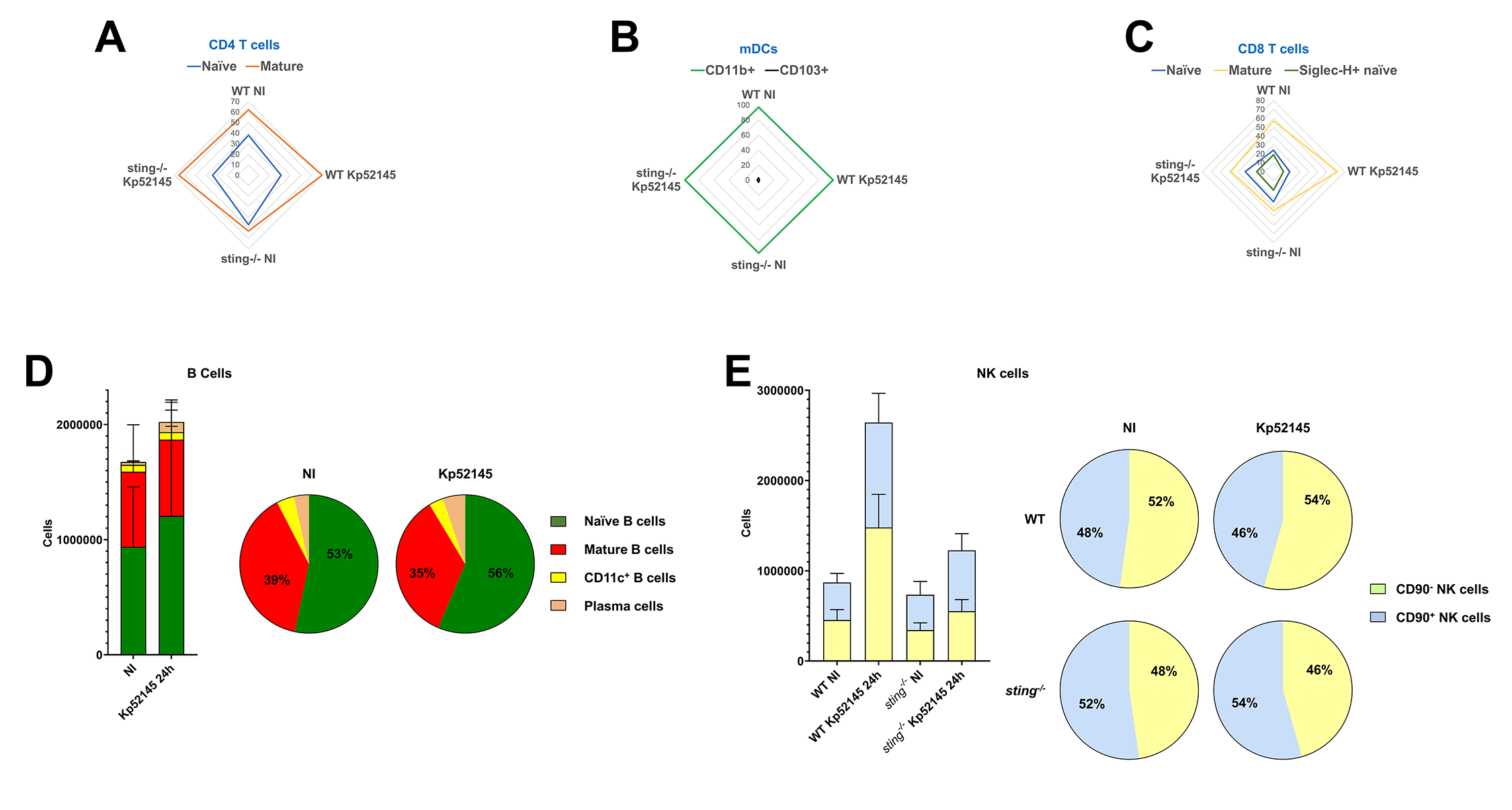

Supplement: S10 Fig — A. Radar plots show the percentage of subpopulations of CD4 T cells in wild-type (WT) and sting-/- non-infected or infected for 24 h with Kp52145. Results are based on data from three mice per group. B. Radar plots show the percentage of subpopulations of mDCs in wild-type (WT) and sting-/- non-infected or infected for 24 h with Kp52145. Results are based on data from three mice per group. C. Radar plots show the percentage of subpopulations of CD8 T cells in wild-type (WT) and sting-/- non-infected or infected for 24 h with Kp52145. Results are based on data from three mice per group. D. Number of cells within each of the subpopulations of B cells in the lungs of sting-/- mice non-infected (NI) or infected for 24 h with Kp52145. Results are based on data from three mice per group. E. Number of cells within each of the subpopulations of NK cells in the lungs of wild-type (WT) and sting-/- non-infected (NI) or infected for 24 h with Kp52145. Results are based on data from three mice per group. (TIF) [file ppat.1011900.s010.tif]
