# Supplementary material for: In vivo single-cell high-dimensional mass cytometry analysis to track the interactions between Klebsiella pneumoniae and myeloid cells
Source: PLoS Pathog. 2024 Apr 5;20(4):e1011900. doi: 10.1371/journal.ppat.1011900 (PMC11023633; doi:10.1371/journal.ppat.1011900)
Supplement: S1 Table — (DOCX) [file ppat.1011900.s011.docx]

**Table S1. Antibodies used for Bac-CyTOF.**

| **CHANNEL-ELEMENT** | **MURINE LUNGS**  **Marker** (Clone) | **SUPPLIER** | **REFERENCE** | **PANEL** |
| --- | --- | --- | --- | --- |
| **103Rh** | **DNA / Live-Dead** | Standard BioTools | 201103B | - |
| **^110^Cd** | ***Klebsiella pneumoniae*** (Rabbit polyclonal) | Abcam | ab20947 | Membrane |
| **^141^Pr** | **Ly-6G** (1A8) | Standard BioTools | 3141008B | Membrane |
| **^143^Nd** | **CD172a / SIRPa** (P84) | BD | 552371 | Membrane |
| **^144^Nd** | **CD45R / B220** (RA3-6B2) | Standard BioTools | 3144011B | Membrane |
| **^145^Nd** | **CD4** (RM4-5) | Standard BioTools | 3145002B | Membrane |
| **^146^Nd** | **F4/80** (BM8) | Standard BioTools | 3146008B | Membrane |
| **^147^Sm** | **CD45** (30-F11) | Standard BioTools | 3147003B | Membrane |
| **^148^Nd** | **CD11b / Mac-1** (M1/70) | Standard BioTools | 3148003B | Membrane |
| **^149^Sm** | **CD19** (6D5) | Standard BioTools | 3149002B | Membrane |
| **^150^Nd** | **CD24** (M1/69) | Standard BioTools | 3150009B | Membrane |
| **^151^Eu** | **CD25 / IL2RA** (3C7) | Standard BioTools | 3151007B | Membrane |
| **^152^Sm** | **CD3ε** (145-2C11) | Standard BioTools | 3152004B | Membrane |
| **^153^Eu** | **CD274 / PD-L1** (10F.9G2) | Standard BioTools | 3153031B | Membrane |
| **^154^Sm** | **TER-119** (TER-119) | Standard BioTools | 3154005B | Membrane |
| **^155^Gd** | **CD103** (2E7) | BioLegend | 121402 | Membrane |
| **^156^Gd** | **CD90.2 / Thy-1.2** (30-H12) | Standard BioTools | 3156006B | Membrane |
| **^158^Gd** | **IL-10** (JES5-16E3) | Standard BioTools | 3158002B | Intracellular |
| **^159^Tb** | **TCRgd** (GL3) | Standard BioTools | 3159012B | Membrane |
| **^160^Gd** | **CD62L** (MEL-14) | Standard BioTools | 3160008B | Membrane |
| **^161^Dy** | **iNOS** (CXNFT) | Standard BioTools | 3161011B | Intracellular |
| **^162^Dy** | **Ly-6C** (HK1.4) | Standard BioTools | 3162014B | Membrane |
| **^163^Dy** | **Siglec H** (551) | BioLegend | 129602 | Membrane |
| **^164^Dy** | **LAP-TGFb** (TW7-16B4) | Standard BioTools | 3164014B | Membrane |
| **^165^Ho** | **Foxp3** (FJK-16s) | Standard BioTools | 3165024A | Intracellular |
| **^166^Er** | **CD192 / CCR2** (475301R) | R&D Systems | MAB55381R-100 | Membrane |
| **^167^Er** | **CD335 / NKp46** (29A1.4) | Standard BioTools | 3167008B | Membrane |
| **^168^Er** | **CD8a** (53-6.7) | Standard BioTools | 3168003B | Membrane |
| **^169^Tm** | **MerTK** (Goat polyclonal) | R&D Systems | AF591 | Membrane |
| **^170^Er** | **NK1.1** (PK136) | Standard BioTools | 3170002B | Membrane |
| **^171^Yb** | **CD44** (IM7) | Standard BioTools | 3171003B | Membrane |
| **^172^Yb** | **CD86 / B7-2** (GL1) | Standard BioTools | 3172016B | Membrane |
| **^173^Yb** | **CD117/c-kit** (2B8) | Standard BioTools | 3173004B | Membrane |
| **^174^Yb** | **MHC-II** (M5/114.15.2) | Standard BioTools | 3174003B | Membrane |
| **^175^Lu** | ***Klebsiella pneumoniae*** (Rabbit polyclonal)  ***Acinetobacter baumannii*** (Rabbit polyclonal) | Abcam  Dr Suzana Salcedo | ab20947  Salcedo laboratory | Intracellular |
| **^176^Lu** | **FcεR1a** (MAR-1)  **DNA / Cell identification** | Standard BioTools  Standard BioTools | 3176006B  201192B | Membrane |
| **^191^Ir** |  |  |  | - |
| **^193^Ir** | **CD11c** (N418) | Standard BioTools | 3209005B | Membrane |
