# Supplementary material for: In vivo single-cell high-dimensional mass cytometry analysis to track the interactions between Klebsiella pneumoniae and myeloid cells
Source: PLoS Pathog. 2024 Apr 5;20(4):e1011900. doi: 10.1371/journal.ppat.1011900 (PMC11023633; doi:10.1371/journal.ppat.1011900)
Supplement: S2 Table — (DOCX) [file ppat.1011900.s012.docx]

**Table S2. Immune populations.**

| **POPULATION** | **CLUSTER** | **SUBSET** | **MARKERS** |
| --- | --- | --- | --- |
| B cells | 2 | Mature B cells | **CD19^+^** **MHC-II^+^** B220^+^ |
|  | 3 | CD11c^+^ B cells | **CD19^+^ MHC-II^+^** B220^+^ CD62L^+^ CD11c^+^ |
|  | 11 | Naïve B cells | **CD19^+^ MHC-II^+^** B220^+^ CD62L^+^ |
|  | 23 | Plasma cells | **CD19^+^ MHC-II^+^** |
| CD4 T cells | 1 | Naïve CD4 T cells | **CD90^+^ CD3^+^ CD4^+^** CD62L^+^ |
|  | 6 | Mature CD4 T cells | **CD90^+^ CD3^+^ CD4^+^** |
| CD8 T cells | 4 | Naïve CD8 T cells | **CD90^+^ CD3^+^ CD8^+^** CD62L^+^ |
|  | 7 | Mature CD8 T cells | **CD90^+^ CD3^+^ CD8^+^** |
|  | 19 | Siglec-H^+^ naïve CD8 T cells | **CD90^+^ CD3^+^ CD8^+^** CD62L^+^ Siglec-H^+^ |
| γδ T cells | 20 | γδ T cells | **CD90^+^ CD3^+^ TCRgd^+^** |
| NK cells | 17 | CD90^-^ NK cells | **NKp46^+^** **NK1.1^+^** |
|  | 21 | CD90^+^ NK cells | **NKp46^+^** **NK1.1^+^** CD90.2^+^ |
| ILCs | 18 | ILCs | **CD90^+^ CD3^-^ NKp46^-^** |
| Alveolar macrophages | 5 | Alveolar macrophages | **SIRPa^+^ MerTK^+^ CD11c^+^ CD11b^-^ MHC-II^+^ CCR2^-^** |
| Recruited MDMs | 14 | Recruited MDMs | **SIRPa^+^ MerTK^+^ CD11c^+^ CD11b^-^ MHC-II^+^ CCR2^+^** |
| Interstitial macrophages | 25 | Interstitial macrophages | **SIRPa^+^ MerTK^+^ CD11b^+^ MHC-II^+^** |
| Inflammatory monocytes | 10 | PD-L1^+^ inflammatory monocytes | **SIRPa^+^ MerTK^-^ CD11b^+^ MHC-II^-^ Ly6C^+^ CD24^-^** PD-L1+ |
|  | 12 | PD-L1- inflammatory monocytes | **SIRPa^+^ MerTK^-^ CD11b^+^ MHC-II^-^ Ly6C^+^ CD24^-^** PD-L1- |
| Resident monocytes | 9 | Resident monocytes | **SIRPa^+^ MerTK^-^ CD11b^+^ MHC-II^-^ Ly6C^-^ CD24^-^** |
| Myeloid dendritic cells | 22 | CD11b dendritic cells | **CD11c^+^ MHC-II^+^ MerTK^-^** CD11b^+^ |
|  | 28 | CD103 dendritic cells | **CD11c^+^ MHC-II^+^ MerTK^-^** CD103^+^ |
| Plasmacytoid dendritic cells | 26 | Plasmacytoid dendritic cells | **CD11c^+^ MHC-II^+^ MerTK^-^ Siglec-H^+^** |
| Eosinophils | 16 | Eosinophils | **SIRPa^+^ MerTK^-^ CD11b^+^ MHC-II^-^ Ly6G^-^ Ly6C^+^ CD24^+^** |
| Neutrophils | 8 | Siglec-H^+^ PD-L1^-^ CD86^+^ neutrophils | **SIRPa^+^ MerTK^-^ CD11b^+^ MHC-II^-^** **Ly6G^+^ Ly6C^+^ CD24^+^** Siglec-H^+^ PD-L1^-^ CD86^+^ |
|  | 24 | Siglec-H^-^ PD-L1^-^ CD86^-^ neutrophils | **SIRPa^+^ MerTK^-^ CD11b^+^ MHC-II^-^ Ly6G^+^ Ly6C^+^ CD24^+^** Siglec-H^-^ PD-L1^-^ CD86^-^ |
|  | 30 | Siglec-H^+^ PD-L1^-^ CD86^-^ neutrophils | **SIRPa^+^ MerTK^-^ CD11b^+^ MHC-II^-^ Ly6G^+^ Ly6C^+^ CD24^+^** Siglec-H^+^ PD-L1^-^ CD86^-^ |
|  | 31 | Siglec-H^+^ PD-L1^+^ CD86^+^ neutrophils | **SIRPa^+^ MerTK^-^ CD11b^+^ MHC-II^-^ Ly6G^+^ Ly6C^+^ CD24^+^** Siglec-H^+^ PD-L1^+^ CD86^+^ |
|  | 33 | Siglec-H^+^ PD-L1^+^ CD86^-^ neutrophils | **SIRPa^+^ MerTK^-^ CD11b^+^ MHC-II^-^ Ly6G^+^ Ly6C^+^ CD24^+^** Siglec-H^+^ PD-L1^+^ CD86^-^ |
|  | 34 | Siglec-H^-^ PD-L1^+^ CD86^-^ neutrophils | **SIRPa^+^ MerTK^-^ CD11b^+^ MHC-II^-^ Ly6G^+^ Ly6C^+^ CD24^+^** Siglec-H^-^ PD-L1^+^ CD86^-^ |
