# Supplementary material for: In vivo single-cell high-dimensional mass cytometry analysis to track the interactions between Klebsiella pneumoniae and myeloid cells
Source: PLoS Pathog. 2024 Apr 5;20(4):e1011900. doi: 10.1371/journal.ppat.1011900 (PMC11023633; doi:10.1371/journal.ppat.1011900)
Supplement: Attachment — Submitted filename: Reviewers comments.docx [file ppat.1011900.s013.docx]

**Reviewer #1:**

This work by Calderon-Gonzalez et al. describes the use of CyTOF with the addition of a bacterial marker (they term Bac-CyTOF) to dissect the immune landscape of a specific bacterial infection in a given niche over time (in this case, Klebsiella pneumoniae in the lung). The authors demonstrate the relative milieu of inflammatory cells over time in the lungs of infected mice. They compare mouse lungs infected with wildtype vs. T6SS-deficient bacteria and find differences in monocytic/macrophage populations. They also look at an additional Klebsiella strain and an Acinetobacter strain and identify some differences in immune cell makeup of the lung at different timepoints. Finally, in a series of elegant experiments, the authors show that the absence of STING results in less PDL1-expressing myeloid cells and a less immunosuppressive environment in the lung. At times the writing is extremely descriptive and dense, but the authors do an admirable job with concluding paragraphs at the end of each results section highlighting the interpretation and importance of their findings. The authors also note in the Discussion that while Bac-CyTOF seems daunting at first, this technique can be powerfully applied after a short learning curve. Overall, this technology seems like a powerful tool for the field, especially when paired with multiple bacterial antibodies allowing for dissection of complicated polymicrobial infections.

We thank the reviewer for this positive evaluation of our work, noting the novelty and the phenomenal applications in other infection contexts.

Some additional clarifications could further strengthen this work:

All comments were well taken, and we have acted as follows.

-The authors describe the relative proportion of immune cells in the lung in many different figures (expressed as %CD45 cells) at many different timepoints. However, they fail to show the true number of each cell type (not just percentage) at each timepoint. For example, the total number of inflammatory cells could change over time and thus while the %CD45 may change, the true number of actual cells total may be constant (or vice versa). Providing absolute cell number (in addition to % totals) could help alleviate these concerns.

As suggested, now we depict the absolute number of cells in all figures. Please note that the conclusions of the study have not changed in terms of decrease/increase population of cells, as well as the dynamic of the different populations over time, and their infection status.

-Are the Klebsiella interactions with more effective killing cells by definition more transient, thus it appears like there are fewer by CyTOF but this is because they are doing their job. Whereas the less active cells have longer interactions with Kleb because they aren’t as good at killing?

With the current evidence and experimental set up, we cannot address this question. Nonetheless, in the context of infection this could be most likely the case. Our data demonstrates that neutrophils, potent phagocytic cell recruited in high number, do not eliminate *Klebsiella* and Bac-CyTOF experiments revealed high levels of infection of these cells. We have demonstrated that alveolar macrophages have a restrictive role (Dumigan et al EMBO Mol Med 2022 14(12):e16888), and we observed a reduction in their numbers only in mice infected with the virulent Kp52145. This is not the case in mice infected with the *clpV* mutant, KP35, and *A. baumannii*, suggesting that the decrease in the numbers of alveolar macrophages found in mice infected with Kp52145 is not linked to the ability of clearance of the pathogen. At present it remains unknown how infection with virulent *Klebsiella* results in a decrease in the population of alveolar macrophages.

-The addition of the bacterial marker in these CyTOF experiments seems to be the important factor. In this case, the authors use an anti-K2 antibody. Do eukaryotic cells that react with this antibody indicate that bacteria are on the surface, intracellular, or both? More discussion about what a cell positive for this marker truly means would be helpful. Also, do the authors find just bacteria in their atlas too? (K2-positive only small cells)?

Our control experiments shown in supplementary figure 2 demonstrate that the antibodies used in this study do not recognize cells. Considering our gating set-up, selecting CD45^+^ cells, bacteria should be at least attached to the cell surface. However, with our staining conditions in which cells are permeabilized it is also likely that most of the detected bacteria are in fact intracellular. Therefore, the reported bacterial data represents a combination of surface attached and intracellular bacteria. We have done a new experiment (new supplementary figure 7) in which we differentially labelled surface attached bacteria, and the intracellular ones. Please note that most of the bacteria detected are in fact intracellular as we did anticipate.

Analysis of the non-CD45^+^ material, detected bacteria associated (surface attached and intracellular) to most likely epithelial and endothelial cells, and fibroblast. In our panel we do not include any antibody for these cells, but the possibility exits to do so as markers to identify these populations are available. We have also added this new information to the manuscript (supplementary figure 7). Although, we have not followed up these interactions, by reporting these observations we provide idea of the power of the technique. We have briefly discussed this possibility (lines 558-561).

-For all the different infections performed throughout the paper, the authors state the given lung titers in text, but fail to show them in graph form. Including the bacterial burdens over time in the figures would be helpful when considering the immune cellular makeups at those same timepoints.

The requested graph now it is included as supplementary figure 1.

-Line 196, does Figure 1G really show “an increase in the number of PD-L1 cells over time, or just that they are increased relative to non-infected lungs and relatively constant throughout? What about total number (not just percentage)?

Now we do report the absolute numbers (Fig 1G), and it is evident the increase in PD-L1 cells over the non-infected lungs and how they are maintained over time.

Minnor comments

I would mention that Kp52145 has markers of hypervirulence (not just say virulent) to distinguish it from the classical ST258 strain.
-Line 44, “These cells” is confusing.
-Line 49-52, confusing. Break up into two sentences, maybe?
-Line 74, delete “The”
-Line 76, what are “hubs”?
-Line 102-104 stats seem a bit antiquated. Either way, a reference is needed for those numbers.
-Line 258, “higher number” of neutrophils? I can only tell that it’s a higher percentage of neutrophils. You never shared numbers with us. This would be helpful.
-Line 298, not sure “required” is the right word because all Klebs increase the levels of PD-L1 to some degree (even though it’s less in T6SS mutants).

We have edited the manuscript as suggested.

**Reviewer #2:**

In this manuscript, Calderon-Gonzalez et al perform a detailed analysis of the cell types involved in the response to Klebsiella pneumoniae (Kp) infection. The authors develop a system Bac-CyTOF, combining mass cytometry with direct labelling of Klebsiella pneumoniae to detect infected cell types to level of subclusters of immune cell populations. Overall, the atlas produced showing the kinetics of immune cell populations and changes in clusters of cells expressing different markers (i.e. PDL1) is interesting. However, there are several issues that limit the interpretation of some of the data:

We greatly appreciate the reviewer’s work assessing out manuscript. All comments were well taken, and we have acted as follows.

1. Throughout the manuscript, various cell types (or subclusters) are reported to be significantly reduced or increased during infection. However, given the massive influx of neutrophils in this infection model, are there actually changes in the absolute numbers of these other populations or is it only a change as a proportion of CD45+ cells?

Now, we do report the changes in absolute numbers. As it cannot be otherwise, the numbers do refer to the CD45^+^ myeloid cells. Please note that our conclusions have not changed in terms of increase/decrease populations and subpopulations of cells over time, as well as their infection status.

2. As a result of the neutrophil influx after infection, many cell types (B,MDM, interstitial macrophages, CD8s etc.) appear to be <1-2% of the CD45+ population. At 30,000 cells analyzed that represents less than 300 cells being analyzed per cell type. These cells are then divided into subclusters and then further stratified by Kp+ or Kp- (i.e. NK cells 1M). This suggests that the number of cells in the subclusters must be very small for all the low abundance cell types, and even lower for the Kp+ or – grouping. These low numbers reduce confidence in interpreting the subcluster dynamics for cell types beyond neutrophils and inflammatory monocytes (Cell types which are already known to be important in response to Kp in a strain dependent manner). In line with this, plots of subclusters are shown without error bars (apart from the more abundant neutrophils in (1K).

We want to mark that Phenograph unsupervised cluster analysis is based on the integration of multiparameters to report phenotypic similarities within the components (in this case cells) of each cluster. The programme is agnostic to the type/subtype of cell, and the number of elements (cells in this case) per cluster. Therefore, the clustering is not based on number of cells or the type of cell but in the integration of parameters defining communities. It should be noted that in our case, to define the different clusters in non-infected mice, and after 24, 48 and 72 post infection the programme integrated more than 4 million parameters (30,000 cells x 4 conditions x 34 markers). Therefore, we consider that the cluster analysis is robust and in fact integrates a significant higher number of parameters than single cell RNA seq, for example, to define the clusters. Subsequent analysis based on the markers of each cluster led us to assign each cluster to a type of myeloid cells. Please note that, as we have explained in the manuscript and the methods, presence of *Klebsiella* is not used for clustering of cells because this would have prevented the integrative analysis of the non-infected mice. The *Klebsiella* marker is only used in a binary manner to determine presence or absence of the bacterium in the different clusters. The quantitative analysis allows us to determine the percentage and, now also the absolute number of cells, within a population/subpopulation positive for *Klebsiella*.

We do not dispute that the number of cells within some of the clusters could be considered small. However, their effect in the immune landscape could be phenomenal. This is exemplified, for example, by the number of DCs and ILC2s. The number of total lung DCs is less than 2% (and pDCs are less than 0.5%) and the number of ILC2 is well bellow 2%; and despite these numbers they do have a crucial role in innate defence against infections.

Now all the plots include the error bars for all populations/subpopulations.

3. Fig 1K: Do the % of infected neutrophils in different subclusters just correspond to the differing abundance of the subclusters over time or is there selectivity in the uptake? Similarly, if Figure 1L is corrected for the baseline differences in non-neutrophil cell type abundance, would that alter the interpretation?

The four main populations are clusters 34, 33, 31, and 24 (as percentage and absolute numbers). However, bacteria were mostly associated with clusters 34 and 33. Of note, at 48 h post infection cluster 34 is the most abundant however bacteria mostly concentrate in cluster 33. Therefore, whereas the more abundant clusters were those with bacteria, our findings demonstrate there is selectivity in the interaction.

The conclusions of Fig 1L do not change by running the analysis suggested by the reviewer.

4. Is Klebsiella bound by/associated with the different cell types or being internalized? What proportion of Klebsiella is cell associated verse free?

In the way that the samples are processed and analysed, we are detecting surface attached and internalized bacteria to any CD45^+^ because these are the cells we are gating. Therefore, in the analysis reported we do not detect any extracellular bacteria. As noted before, in this revised version we do include new results (supplementary figure 7) in which we differentially label surface attached bacteria, and the intracellular ones. Please note that most of the bacteria detected are in fact intracellular. By analysing the non-CD45^+^ material we have been able to detect *Klebsiella* surface attached to non-CD45^+^ cells (most likely epithelial and endothelial cells, and fibroblasts) and the intracellular ones (the minority). This is consistent with the evidence showing that *Klebsiella* is non-internalized by these cell types (Bengoechea et al FEMS Microbiol Rev 2019 43(2):123-144). In the context of *Klebsiella* lung infections, the evidence demonstrates the presence of extracellular bacteria (we believe the majority), and those associated to myeloid cells (this work, and Dumigan et al EMBO Mol Med 2022 14(12):e16888) (the relative minority in terms of bacterial numbers). However, this population of bacteria associated to myeloid cells is crucial to shape the lung microenvironment to promote infection as we have recently demonstrated by assessing the interaction of *Klebsiella* with different populations of lung macrophages (Dumigan et al EMBO Mol Med 2022 14(12):e16888).

5. Fig 2: Why were the experiments performed only for 48 hours in this case? The authors demonstrate that there are differences in the kinetics of WT vs ClpV infections (based on CFU/gr) and the immune responses to WT Kp over the first 72 hours. The same time course should be used for the ClpV strain.

Our findings establish that the major differences in the atlas occur at the 24 h and 48 h; at 72 h the results are like those found at 48 h. These findings dictated the time points to be tested when assessing the atlas of the mice infected with the *t6ss* mutant.
6. Fig 2: Why would ClpV be higher at 24 hours? Wouldn’t the higher bacteria load explain increased neutrophil recruitment at that timepoint rather than any difference as a result of the T6SS?

We can only speculate why the loads of the *clpV* were higher than those of the wild type at 24 h. Our working hypothesis is that the host detects the activation of the T6SS resulting in an initial protective inflammatory response. Therefore, lack of the T6SS initially favours bacterial survival. In any case, it should noted that *Klebsiella* encodes a number of non-redundant immune evasins (Bengoechea et al FEMS Microbiol Rev 2019 43(2):123-144), explaining why the wild-type strain is able to counteract this initial protective response. Bac-CyTOF analysis have demonstrated the contribution of the T6SS to shape the immune atlas including the levels of the immune checkpoint PD-L1, and the interaction of *Klebsiella* with cells controlling the infection. This evidence is consistent with the observation that over time the *t6ss* mutant is not able to flourish in the tissue as the wild-type strain. We have chosen not to discuss this as it remains just a working hypothesis.

Our results show the increased number of neutrophils in the mice infected with the *t6ss* mutant compared to mice infected with the wild-type strain. We kindly disagree with the reviewer’s point negating the contribution of the T6SS. Of note, in the other two infections with KP35 and *A. baumannii*, the bacterial loads were two-logs lower than those found in the *t6ss* mutant infected mice, but the levels of neutrophils were similar, suggesting that in this infection model bacterial loads alone cannot explain differences in the levels of neutrophils.

7. Fig 2AB: Given the decrease in % of neutrophils of the CD45+ population at 48hrs, wouldn’t the % of the other populations like inflammatory monocytes (the second most abundant) be larger even if there was no actual difference in the recruitment or function of the inflammatory macrophages themselves. (related to comment 1 above).

Now we report the absolute numbers of immune cells. In agreement with our previous observations there is a significant increase in the number of inflammatory monocytes.

8. Lines 272-276: Cluster 17 appears to be very minor in the case of Wt Kp infection (Fig 1M). Are the cluster numbers being re-mapped with the ClpV strain? If so, the data should be analyzed together to enable direct comparison of the subclusters between the infections (as plotted in S7). If the data can't be analyzed together because it was acquired on different days, the infections should be repeated and compared directly.

Data were analysed together, allowing direct comparisons of the results.

9. Fig 2I: the authors show a difference in alveolar verse interstitial macrophages at 24 hours between ClpV and Wt klebsiella which normalizes by 48 hours – by which point the WT Kp have replicated to the same CFU/gr as the ClpV reach in 24 hours. This suggests that bacterial load could play an equally important role as the T6SS in determining cell populations and uptake (which seems to correlate with which cells are present in 2B).

On may argue that it is the relative differences in the number of alveolar macrophages and interstitial macrophages what dictates the bacterial interactions. We want also to mark that at 24 h post infection, and despite the high number of *clpV* bacteria, this strain selectively localizes within alveolar macrophages and not interstitial macrophages. Moreover, when considering for each strain the total number of alveolar macrophages we found clear differences between genotypes that also were sustained over time (new Fig 2J).

10. Fig 5: STING experiments: Given the documented role for the microbiota to influence immune system development and function in the gut and systemically (including in the lung), are the changes in the response to Kp infection in Sting KO observed with littermate WT and STING KO mice?

The experiments were not done with littermate wild-type mice; however, the mice were co-housed to normalize microbiota. Nonetheless, and as rightly pointed out by the reviewer, reports indicate that absence of STING results in changes in the gut microbiome (Canesso et al Mucosal Immunology 11:820–834 (2018)). It was observed a reduction in the *Allobacolum* (Firmicutes) and *Bifidobacterium* (Actinobacteria) groups along with an increase in *Disulfovibrio* (Proteobacteria) bacteria. In contrast, there were no significant changes in *Bacteroides/Bacteroidetes*. Importantly, these members of the microbiome are those controlling protection against *Klebsiella* in the pneumonia mouse model (Sequeira et al Nature Microbiology. 5(2): 304–313 (2020(). Altogether, this evidence suggests that differences in the gut microbiome could not explain our findings challenging *sting^-/-^* mice. On-going research in the laboratory is shedding light into the STING-dependent signalling promoting *Klebsiella* infection. The work is illustrating the importance of STING signalling to counteract cell intrinsic immunity upon *Klebsiella* infection.

11. Fig 5: STING experiments: similar to the ClpV mutant (point 5 above), why is only a single timepoint being examined? It is hard to measure clearance with a single timepoint that in other contexts is being used as a measure of initial infection. What happens at 48 and 72 hours?

A 24 h time point is biological relevant in the case of *Klebsiella*-induced pneumonia to assess clearance. We would like to refer the reviewer to the following studies from Mobley’s and Bachman’s labs (Bachman et al [mBio.](https://www.ncbi.nlm.nih.gov/pmc/articles/PMC4462621/) 2015 May-Jun; 6(3): e00775-15; Vornhagen et al PLoS Pathog. 2019 15(8):e1008010; Holmes et al [Infect Immun.](https://www.ncbi.nlm.nih.gov/pmc/articles/PMC9302095/) 2022 90(7): e00224-22.), Hauser lab (Agard et al [Infect Immun.](https://www.ncbi.nlm.nih.gov/pmc/articles/PMC6529660/) 2019 87(6): e00871-18.) probing wild-type mice, and the work from a number of labs including ours challenging mice knock-out for innate receptors and adaptors (for example Cai et al [J Immunol. 2009 183(10): 6629–6638.](https://www.ncbi.nlm.nih.gov/entrez/eutils/elink.fcgi?dbfrom=pubmed&retmode=ref&cmd=prlinks&id=19846873) ; van Lishout et al J Infect Dise 2012 9:1415–1423; van Lishout et al [PLoS Pathog.](https://www.ncbi.nlm.nih.gov/pmc/articles/PMC4177915/) 2014 10(9): e1004368.; Suresh et al [JCI Insight.](https://www.ncbi.nlm.nih.gov/pmc/articles/PMC6962028/) 2019 4(23): e131195.; Feriotti et al Cell Rep. 2022 40(6):111167; Wieland et al Eur Respir J 2011 37(4):848-57).

12. Fig 5: The STING experiments appear to have been performed at a different time compared to the WT to which they are compared (same data is shown in Fig 5 as in Fig 1). The responses of wildtype and sting mouse lines should be compared (using littermates), during infections performed with the same inoculum and analyzed together. The changes in cell types (Fig 5A,B) and clusters are relatively minor, and could result from a different inoculum/starting bacterial load – this should be controlled for.

The Bac-CyTOF experiments and the assessment of *Klebsiella* survival were done different times, and therefore using different inoculi. However, for each type of experiment both mouse genotypes were tested and analysed at the same time. In our hands, the intranasal infection model is highly reproducible. The method used to prepare the inoculum (describe in detail in the methods section) is standardized in the laboratory with no more than 5% variation in bacterial dose between different days.

Minnor comments

13. It would be helpful for the reader if the cluster colors in pie charts consistently mapped to the cultures in the tSNE. Furthermore, in some cases, colors in legends do not match colors in pie charts
14. Fig 1D: The percentages in this panel appear to be mislabeled.
15. Throughout the figures, it would be helpful for the reader to have labels describing the cell type (immature B cell) rather than just labelling bar/pie charts with non-sequential cluster numbers.

We have followed the reviewer’s advice and edited the manuscript as suggested.

**Reviewer #3:**

This is a very interesting study that utilises single cell mass cytometry to evaluate the interactions between Klebsiella pneumoniae and host cells. The use of MS Cytof to evaluate host-pathogen interactions (which the authors coin the term Bac-CyTOF) is novel and will be of substantial value to the infection biology field. This work contributes to the knowledge on the immune evasion phenotype of k. pneumoniae.
A key strength of this work is the ability to identify the multiple immune cell types that interact and are infected by K. pneumoniae. Moreover the ability to hone in on specific cell populations, e.g. non-neutrophils, is informative. In addition, the availability of transgenic mouse lines from the Bowie lab (not authored) to examine specific pathways is another strength.
The identification of PDL1 activation associated with immune cell exhaustion is very interesting and will be of relevance beyond the klebsiella field.
Overall the paper shows that mice infected with their Kp strain show a decrease in populations of the cells that are required to clear infections or deactivation of cells necessary for clearance.

We thank the reviewer for her/his positive comments on our work, noting the novelty and potential impact in the field. We do appreciate the helpful insights, and we have acted as follows.

One issue is the biological relevance of some of the differences in cell numbers identified. While differences in the numbers of different cell types (alveolar macrophages in infected and non-infected mice; or those infected with different bacterial strains or species) have been shown and are statistically significant, it is not clear whether they are biologically significant.

To illustrate the potential relevance, we will comment on the alveolar macrophages. We have recently demonstrated that a ~ 50% reduction in the number of alveolar macrophages results in a 3-log difference in the bacterial loads of mice infected with Kp52145 (Dumigan et al EMBO Mol Med 2022 14(12):e16888). Here, we report similar differences or even larger between non-infected and Kp52145-infected mice for 24 h (~ 70% reduction in alveolar macrophages in the infected mice), whereas there is a 90% increase in the number of alveolar macrophages in the mice infected with KP35 and *A. baumannii* compared to mice infected with Kp52145 for 24 h. It is certain that the significant increase in the number of alveolar macrophages as well as the increased number of neutrophils found in KP35 and *A. baumannii* infected mice account for the clearance of these two pathogens. Please note, that in the case of neutrophils we have detected that cluster 34 accounts for more than 80% of the neutrophils found in KP35 and *A. baumannii* infected mice whereas this cluster represents only 39% of the neutrophils found in Kp52145-infected mice at 48 and 72 h post infection. At these time points, the bacterial loads of Kp52145 were 3 and 4-logs higher than those of KP35 and *A. baumannii*.

It is remarkable that the comparison of the virulent Kp strain and the clpV mutant strain shows that the majority of differences are most apparent at 24 hours rather than at 48 hours, suggesting that the presence of clpV / T6SS is accelerating the effect. Can the authors comment on the kinetics of the interactions? And the fact that the differences are relatively short-term?

We want to mark that the role of the T6SS in *Klebsiella* virulence is still poorly understood, making difficult to provide any explanation for the observed phenotype. Our work starts to unveil that the T6SS belongs to the portfolio of weapons exploited by *Klebsiella* to counteract the innate immune system. Here, we have uncovered the contribution to the upregulation of the immune checkpoint PD-L1 and to shape the interaction with lung macrophages. In our recent study, we demonstrated the role targeting the mitochondria to blunt inflammation (sa Pessoa et al Nat Commun 2023 14(1):871). We lack understanding of the effector(s) responsible for the phenotype in vivo as well as whether there are spatial-temporal elements that need to be considered. Future complex studies beyond the scope of this work are warranted to address these questions.

Two strains are compared, a virulent strain, Kp52145 and another AMR strain. While there are some differences observed between these 2 strains, some of the statements in the discussion, e.g. suggest that this is a broader effect of virulent strains generally. The authors should qualify these statements.

The comparison between these strains refers to their ability to infect mice. In this model, KP35 cannot be considered a virulent strain. This is a well-known feature of most clinical *Klebsiella* strains even if they are MDR. Now we clearly indicate that the results refer to a hypervirulent strain whereas KP35 is cleared by the mice.

Differences are observed in the myeloid cell interactions between the virulent strain Kp52145 and the carbapenem resistant strain Kp35. Are these merely strain-specific differences or are there real differences between "virulent strains" and other strains? The authors should attempt to explore additional strains to verify their claims using alternative methods to determine how general these observations are.

While this is a valid point considered the genetic diversity of *K. pneumoniae*, we believe the suggested experiment is beyond the scope of this work. Here, we have tested a *Klebsiella* strain that it is cleared by the mice over time, KP35, and a strain of other species, *A. baumannii*, that it is also cleared by the mice. In both cases, we have observed remarkable similarities in the atlas as well as clear differences with the atlas found in mice infected with Kp52145, the *Klebsiella* strain that it is not clear by the mice. We believe this evidence illustrates well our point of the differences between infections with different outcome in vivo. We have noted in the text that Kp52145 is a hypervirulent strain. KP35 is attenuated in the pneumonia mouse model (line 360-362).

Whether by testing different *Klebsiella* strains we might find differences in the described atlases shall the subject of a new study that addresses a different question “*how does Klebsiella genetic diversity shapes the described atlases?*” A tool to address this question could be the recently described collection of 100 *Klebsiella* strains established by the US Army (Martin et al Microb Genom. 2023; 9(5): mgen000967). We are currently investigating the interaction of these strains with the innate system, and eventually in the future we will use Bac-CyTOF to further analyse their virulence.

Minnor comments

1. The authors refer to the interaction (singular) between myeloid cells and K. pneumoniae. however it is clear that there are multiple interactions and the plural form "interactions" should be used in the title and throughout the text.

We have changed the text accordingly.

2. It is not clear how the authors establish that the cells are actually infected cells. Can the authors confirm that the host cells referred to are actually infected and not associated with the bacterial cells ? e.g. Fig 1 A and Fig 1K; Fig 2I and 2J etc.

In our gating method, we have selected CD45^+^ events (cells), and then subsequently asked whether they are positive for the *Klebsiella* or *Acinetobacter* antibodies. Therefore, bacteria need to be at the very least surface attached. However, in the way we do the staining the anti-bacteria antibody will also have access to intracellular bacteria. Therefore, we are detecting a combination of attached, and internalized bacteria. New experiments, reported in supplementary figure 7, were caried out to identify attached versus intracellular bacteria. Indeed, we detect a combination of attached and intracellular bacteria, being the latter the predominant.

3. In some cases, e.g. Fig 5B Lines 431 to 434, differences in levels of cells are reported which look fairly comparable. Are these biologically relevant? Can the authors report them as "fold-differences" to highlight whether they are actually different? .

We have added this information in the text (now lines 457-460).

4. Line 171 to 5. What is the source of the B cells being shown in Figure S4A? this section needs clarification. Which data are "these data" in line 173.

These B cells are the same ones used for the cluster analysis. The diffusion maps analyses utilize the single-cell data and the cluster to infer trajectories within the type of cells across the different clusters.

5. Figures and associated text.
While the breadth of cells that are identified is a key strength of this manuscript, the presentation of the results could be improved. It would be useful if the keys were more informative in the figures. For example B-cell clusters that are presented in Fig 1C and 2C, are identified in the text as being Naïve or mature etc, in the text, but the figure only shows cluster 11, cluster 2. The same can be said for Figs 1D & 2D and the neutrophil clusters. The keys in figure 1 and 2 could be more informative.

We have tried out best to improve the presentation to facilitate the readers’ assessment of the work. For example, note figures 1D, 2C, 3D, 4D, 4H.

6. Comparisons are made between different cell populations across more than one figure. It would greatly aid the readers if the figure being compared is also identified within the main text, so that the reader doesn't have to work it out for themselves. By way of example Figure 5I is compared with Figure 3D, but it is not immediately obvious in the main text as to which of the previous figures is the one to compare. Related to this, the reader is required to flick between various figures to visualise what the authors are demonstrating, it would be useful if the WT data was shown as a comparison in figure 2, for example.

When possible, we have tried our best to follow the reviewer’s advice (for example figs 5A and 5B and 2J). When the differences are dramatic, for example, the dominance of neutrophils cluster 34 in mice infected with KP35 and *A. baumannii*, and the infection of B cells only in *sting^-/-^* mice, we have kept the data set in different figures

7. Figure 4D - it appears that the A. baumannii strain is concentrated in clusters corresponding to neutrophils predominantly and the interaction with alveolar macrophages is very small in comparison (looks to be <5%).
Indeed, this is the case. In the case of *Acinetobacter* the vast majority of bacteria localizes with neutrophils.

8. Similarly the differences in alveolar macrophages in the sting-/- infected mice versus WT mice also seem to be minimal
Now we show the number of alveolar macrophages as an inset (refer to new Fig 5B); data shows the statistically significant differences between mouse genotypes (lines 457-460).

9. There are several grammatical errors that impair smooth reading. I counted 9 grammatical errors in the abstract alone. The manuscript should be rigorously proof read by all authors before resubmission.

We have done our best to correct all the errors. All authors have proofread the submitted version.
